# The Effects of Zinc Supplementation on Growth, Diarrhea, Antioxidant Capacity, and Immune Function in Holstein Dairy Calves

**DOI:** 10.3390/ani13152493

**Published:** 2023-08-02

**Authors:** Junhao Liu, Fengtao Ma, Allan Degen, Peng Sun

**Affiliations:** 1 State Key Laboratory of Animal Nutrition, Institute of Animal Science, Chinese Academy of Agricultural Sciences, Beijing 100193, China; 2 Desert Animal Adaptations and Husbandry, Wyler Department of Dryland Agriculture, Blaustein Institutes for Desert Research, Ben-Gurion University of the Negev, Beer Sheva 8410500, Israel

**Keywords:** calf health, growth performance, plasma trace elements, zinc oxide, zinc proteinate

## Abstract

**Simple Summary:**

Diarrhea is the most common disease in dairy calves and occurs frequently during the first month of life. It affects animal welfare and causes substantial economic losses. Zinc (Zn) is an essential nutrient element that can reduce the incidence of diarrhea. This study was designed to examine the effects of supplementary Zn proteinate (ZnPro, an organic zinc source) and Zn oxide (ZnO, an inorganic zinc source) on growth performance, diarrhea, antioxidant capacity, immune function, and mineral element concentrations of dairy calves during the first 28 days of life. Both ZnPro and ZnO reduced the bouts of diarrhea and promoted the immune function of calves, but ZnPro improved the growth performance and antioxidant capacity to a greater extent than ZnO. It was concluded that ZnPro is a better zinc source to improve calf health and performance than ZnO.

**Abstract:**

The current study examined the effects of supplementary zinc proteinate (ZnPro) and zinc oxide (ZnO) on growth performance, diarrhea, antioxidant capacity, immune function, and mineral element concentrations of calves aged 1 to 28 days. A total of twenty-four newborn calves were divided randomly into 3 groups (n = 8; 2 males and 6 females per group), and each received: 0 mg/d Zn (CON), 627 mg/d ZnPro (80 mg/d Zn; ZnPro group), and 101 mg/d ZnO (80 mg/d Zn; ZnO group). The calves received the additive in their milk during the first 28 days of life. Compared with the CON group: ZnPro and ZnO improved average daily gain (ADG) and decreased the feed:gain ratio (FGR) between days 1 and 14 (*p* < 0.05), while the ADG increased and FGR decreased only in the ZnPro group between days 1 and 28 (*p* < 0.05). The incidence of diarrhea decreased (*p* < 0.05) in the ZnPro and ZnO groups between days 15 and 28 as well as days 1 and 28, but decreased (*p* < 0.05) only in the ZnPro group between days 1 and 14. The serum immunoglobulin G (IgG) concentration of the ZnPro and ZnO groups increased on days 14 and 28 (*p* < 0.05). ZnPro supplementation increased serum IgM concentration during the whole study, while ZnO enhanced serum IgM concentration only on day 14 (*p* < 0.05). In the ZnO group, the serum concentration of cytokines interleukin (IL)-10 increased on day 14, while that of IL-1β increased on day 28 (*p* < 0.05). In addition, ZnPro reduced the serum malondialdehyde (MDA) concentration on days 14 and 28 (*p* < 0.05). Both ZnPro and ZnO increased the serum concentrations of alkaline phosphatase (ALP) and metallothionein (MT) on day 14 (*p* < 0.05). With zinc supplementation, plasma Zn concentration increased (*p* < 0.05) on days 14 and 28 of age. We concluded that supplementary ZnPro and ZnO reduced incidences of diarrhea and promoted the immune function, but ZnPro improved the growth performance and antioxidant capacity of Holstein dairy calves to a greater extent.

## 1. Introduction

Keeping calves healthy is the key to promoting the dairy industry. Studies have demonstrated that higher daily gain and healthy and robust calves could enhance their first lactation and lifetime productivity [1,2,3]. This suggests that improving the average daily gain and health of calves during the pre-weaning stage can improve their productivity in the future [4]. Diarrhea is the most common disease in dairy calves and occurs frequently during the first month of life [5]. It can cause scours, general weakness, and ultimately the death of the calf. Consequently, it is particularly important to prevent diarrhea during the first month after birth. Antibiotics have been used to improve animal growth and prevent disease [6], which may result in bacteria developing antibiotic resistance [7]. Therefore, it is crucial to find new safe additives to improve the growth performance and health of livestock, as antibiotics have been banned in many countries [8].

Zinc (Zn) is an essential nutrient element and is involved in many physiological functions in the body [9]. Supplementary Zn has increased growth performance, promoted antioxidant and immune activities, improved intestinal microflora, and reduced the incidence of diarrhea [10,11]. Zinc proteinate (ZnPro) is a novel form of organic Zn prepared from soybean protein isolate and feed-grade inorganic Zn [12]. Zn ions in ZnPro are bound with amino acid ligands, which protect the zinc ions from combining with phytic acid and prevent antagonism between metal ions when absorbed [13,14]. Therefore, organic zinc overcomes the disadvantages of traditional inorganic zinc, such as low bioavailability and the destruction of feed nutrients [15]. Moreover, supplementary ZnPro reduces lipid peroxidase activity and increases humoral immunity and superoxide dismutase (SOD) activity in lamb red blood cells [16].

Bioavailability of organic zinc is greater than inorganic forms, but different zinc sources function in different modes. In a previous study, supplementary ZnPro at 80 mg Zn/day increased the average daily gain (ADG) and immune function, and reduced the incidence of diarrhea in dairy calves during the first 2 weeks of life [17]. Furthermore, Wei et al. [18] reported that supplementary ZnO at 80 mg Zn/d improved ADG in calves aged 1 to 14 days. Of the natural form of zinc, ZnO is the only inorganic form of zinc with these benefits [9]. However, it is unclear if this improvement continues with increasing time of addition and if there is a difference in the impact of the two zinc sources. Therefore, we supplemented calves with ZnPro for 28 days after birth at a daily level of 80 mg Zn/d and determined their ADG, incidence of diarrhea, antioxidant capacity, immune function, and mineral element concentrations in the serum compared with ZnO. The results suggest a basis for the provision of ZnPro as a novel Zn source for newborn calves.

## 2. Materials and Methods

This study was conducted from 1 November 2022 to 21 December 2022 at Hebei Junyuan Farm (Xinle, China). All procedures were approved by the Institute of Animal Science, Chinese Academy of Agricultural Sciences (Beijing, China). All animals in the present study were raised according to the standards established by the Institute of Animal Science, Chinese Academy of Agricultural Sciences (Beijing, China).

### 2.1. Experimental Design, Animals, and Management

Twenty-four newborn, healthy Holstein dairy calves (42.2 ± 1.06 kg) were selected for the study. Each calf was housed individually (1.8 m × 1.4 m × 1.2 m) to avoid cross contamination. The calves were allocated randomly into 3 groups (n = 8, 2 males and 6 females in each group) and each calf received one of the following levels of zinc supplement in its milk: 0 mg Zn/d (CON group); 627 mg/d ZnPro (80 mg zinc/d; ZnPro group); and 101 mg/d ZnO (80 mg zinc/d; ZnO group). These doses were based on previous studies [17,18]. The ZnPro (Q_f_ = 352, purity: 12.76%) was donated by the Mineral Nutrition Research Division, Institute of Animal Science, Chinese Academy of Agricultural Sciences (Beijing, China), while the ZnO (purity: 99%) was purchased from Shanghai Macklin Biochemical Technology Co., Ltd. (Shanghai, China).

The ZnO or ZnPro was mixed with 100 mL milk and fed to the calves on days 1 to 28 after birth. The milk was heated to 60 °C, then cooled to 38 to 39 °C and fed to the calves. The calves were fed from a bottle; each calf received 4 L of colostrum within 1 h of birth, followed by 2.5 L of raw milk three times per day at 06:00, 12:00, and 18:00. At 15 to 28 days of age, the calves received 3 L instead of 2.5 L each time. The calves had free access to starter and water. The nutrient compositions of the milk and starter are presented in Table 1 and Table 2.

### 2.2. Sample Collection

Blood samples were collected from the external jugular vein of each calf using vacutainer tubes, with or without heparin sodium (BD Biosciences, San Jose, CA, USA), before morning feedings on days 15 and 29. Serum and plasma were collected following centrifugation at 3000× *g* for 15 min at 4 °C and then stored at −20 °C for subsequent analyses.

### 2.3. Average Daily Gain

Each calf was weighed before morning feedings on days 1, 15, and 29. ADGs were calculated for these time intervals and for the entire length of the study. Daily intakes of milk and starter were recorded, and the total dry matter intake (DMI), starter DMI, and feed:gain ratio (FGR) were calculated for each calf. 

During the trial period, the feces of each calf were observed twice daily, in the morning and evening, and given a score of 1 to 4, as suggested by Teixeira et al. [19]. Normal and cylindrical feces were scored as 1 point; slight, thin, soft, and tangible feces as 2 points; shapeless stools with high moisture content as 3 points; and liquid, shapeless, watery feces as 4 points. Scores of 3 and 4 for 2 consecutive days were defined as diarrhea, and the incidence of diarrhea was recorded [20].

### 2.4. Immune Status and Antioxidant Status

Serum immunoglobulin A (IgA), immunoglobulin G (IgG), immunoglobulin M (IgM), interleukin-1β (IL-1β), interleukin-10 (IL-10), and interferon-γ (IFN-γ) concentrations were measured using bovine ELISA kits (Wuhan Genomei Technology; Wuhan, China) according to the manufacturer’s instructions.

The activity of serum superoxide dismutase (SOD), the serum concentrations of total antioxidant capacity (T-AOC), and malondialdehyde (MDA) were determined via radioimmunoassays using commercial kits (Beyotime Biotechnology, Shanghai, China) in accordance with the manufacturer’s instructions.

### 2.5. Serum Zinc-Dependent Protein and Plasma Trace Element Concentrations

Serum alkaline phosphatase (ALP) and metallothionein (MT) concentrations were determined using commercial kits (Wuhan Genomei Technology, Wuhan, China).

Concentrations of plasma copper (Cu), iron (Fe), zinc (Zn), calcium (Ca), phosphorus (P), and magnesium (Mg) were measured with inductively coupled plasma optical emission spectroscopy (ICP-OES), according to the Chinese National Standards (GB 5009.268, China, 2016).

### 2.6. Statistical Analysis

The incidence of diarrhea was analyzed using Chi-square test with SAS (version 9.4, SAS Institute Inc., Cary, NC, USA), and ADG, immune indices, antioxidant capacity, zinc-dependent protein indicators, and plasma trace element concentrations were analyzed using one-way ANOVA and Tukey’s method for multiple comparisons. Statistical significance was accepted as *p* < 0.05, and 0.05 < *p <* 0.10 as tended to be significant.

## 3. Results

### 3.1. Growth Performance

There was no difference in the initial weight of calves among the three groups (Table 3). Supplementation with ZnPro and ZnO increased the ADG and decreased the FGR of calves (*p* < 0.05) at 1 to 14 days of age, while only ZnPro supplementation enhanced the ADG and lowered the FGR compared with the CON group at 1 to 28 days of age (*p* < 0.05). The incidence of diarrhea decreased in the ZnPro and ZnO calves during the whole experimental period.

### 3.2. Immune Status and Antioxidant Status

Compared with the CON group, both Zn groups had higher serum concentrations of IgG and IgM content (*p* < 0.05), but only the ZnO group had a higher serum concentration of IL-10 than the CON group at 14 days of age (*p* < 0.05, Table 4). At 28 days of age, ZnPro increased the serum concentration of IgM (*p* < 0.05), whereas ZnO decreased the serum concentration of IL-1β compared with the CON group (*p* < 0.05). In addition, the serum IgG concentrations of the calves in the ZnPro and ZnO groups were higher than in the CON group (*p* < 0.05).

Compared with the CON group, ZnPro calves had lower (*p* < 0.05) serum concentrations of MDA than the CON calves at 14 and 28 days of age (Table 5). However, the ZnO calves had lower (*p* < 0.05) serum concentrations of MDA only at 28 days of age. Both Zn groups had higher serum T-AOC concentrations at 14 days of age (*p* < 0.05).

### 3.3. Serum Zinc-Dependent Protein and Plasma Trace Element Concentrations 

The calves in both the ZnPro and ZnO groups had higher (*p* < 0.05) concentrations of serum ALP and MT at 14 days of age (Table 6). Plasma Zn concentrations at 14 and 28 days of age were higher (*p* < 0.05) in the ZnPro and ZnO calves than in the CON calves (Table 7). 

## 4. Discussion

Growth performance including ADG, feed intake, and FGR are the most important parameters reflecting the health and growth status of calves. Our previous study reported that ZnPro supplementation improved the growth performance of dairy calves during their first two weeks of life [17]. In the present study, supplementary zinc of 80 mg/d from ZnPro improved the ADG and decreased the FGR in calves during the whole experimental period of days 1 to 28, while improvements with ZnO occurred only during1 to 14 days of age. These differences may be due to the organic nature of ZnPro, which facilitates its absorption and utilization in animal tissues and exhibits better growth-promoting effects than inorganic forms [21]. Similar results were observed by Wo et al. [17], who found that ZnPro supplementation of more than 80 mg zinc/day increased ADG and decreased the feed-to-gain ratio during days 1 to14, and its effects were comparable to the same dose of zinc in the form of zinc methionine when supplemented for 28 days.

Diarrhea is the most common disease in calves, occurring frequently in the first month after birth [5]. There are many reasons for calf diarrhea, including incomplete intestinal development, incomplete absorption of nutrients, weak immune function, and stress from cold or hot environments [22]. For decades, zinc has been used as an anti-diarrheal agent to prevent and treat diarrhea in infants and children, as well as in animals. Our research team focused on investigating the effect of a low dose of Zn supplementation in reducing the incidence of diarrhea in dairy calves. Supplementation with organic Zn, such as Zn-methionine and ZnPro, at 80 mg Zn/d effectively alleviated diarrhea in calves during the first two weeks of life [17,20]. Consistent with these results, in the current study, ZnPro and ZnO reduced the occurrence of diarrhea at 1 to 28 days of age. Notably, ZnO did not reduce the occurrence of diarrhea in calves during 1 to 14 days of age, but it had an anti-diarrheal effect after supplementation for 28 days. 

In mammals, immunoglobulins (IgA, IgM, and IgG) represent the main components of humoral immunity, protecting animals against pathogen attacks by recognizing invading pathogens and activating multiple immune responses [23,24]. Zinc acts as an immunostimulant agent that enhances cellular and humoral immune system responses [25]. In the current study, ZnPro and ZnO increased serum IgM and IgG concentrations in calves at 14 days of age, and ZnPro also elevated IgM concentration at 28 days of age, which adds one more biological advantage to ZnPro, as IgM is considered the first line of host defense against infections and also plays an important role in immune regulation and immunological tolerance [26]. In this study, the findings suggest that zinc supplementation improves immune capacity and, thereby, reduces the incidence of diarrhea. Similarly, Wo et al. reported that the serum concentrations of IgG and IgM increased on day 28 by supplementing calves with ZnPro during the first month after birth [17]. Furthermore, Zn regulates the chemotaxis and phagocytosis of polymorphonuclear leukocytes through the NF-κB signaling pathway, inhibits the production of inflammatory IL-1β and TNF-α [27], regulates Th lymphocyte balance by regulating the release of peripheral blood monocyte INF-γ, and affects IL-10 and TNF-α expressions to improve immune function [27,28]. In the present study, only ZnO reduced serum IL-1β level of 28-day-old calves compared with the CON group. This is similar to a previous study in growing rabbits [11], in which supplementary 50 mg/d ZnPro did not affect serum IL-1β concentration. IL-10 has protective effects on the intestinal mucosal barrier [29]. Ram et al. demonstrated that the intravenous administration of trace element mixtures (Zn concentration at 40 mg) increased serum IL-10 levels in diarrheal calves [30]. Consistent with this report, the serum IL-10 concentration in the current study increased in the ZnO-fed calves at 14 days of age.

Zinc can protect cells from oxidative damage, such as reactive oxygen species, free radicals, and other substances, by binding with thiol groups [27,31]. Reactive oxygen species (ROS), which are generated during normal cellular respiration, can be scavenged through the free radical scavenging system [32]. T-AOC is the most common antioxidant biomarker, which reflects the propensity to scour [33]. In our study, the T-AOC concentration increased with Zn addition in the 14-day-old calves, which indicates that Zn has the ability to raise the antioxidant. The MDA is an end-product of lipid peroxidation, regarded as a marker of oxidative stress and antioxidant status [34]. Previous studies reported that dietary Zn can decrease MDA concentration and increase T-AOC concentration in the serum of ruminants [18,35]. Consistent with previous reports, in the present study, supplementary ZnO decreased the serum concentration of MDA on d 28, while supplementary ZnPro decreased the serum concentration of MDA on d 14 and 28. As antioxidant capacity increased, the ability of the calves to cope with stress increased, which was another major reason for diarrhea prevention in calves [36]. 

Zinc can be transferred into the circulatory system through MT during intestinal absorption, so MT can regulate Zn balance and metabolism in the body [11,37]. In addition, an MT molecule can combine with seven Zn ions to scavenge ROS in liver and bone marrow cells; its scavenging ability of ·OH is 300-fold greater than GSH-Px [38]. Therefore, the MT level can reflect both Zn transport and antioxidant capacity in the body. In the present study, the serum MT concentration increased with both Zn sources, which was consistent with the trend displayed with plasma Zn concentration. The high MT content indicated that the antioxidant capacity of calves in the two groups was enhanced. In addition, Zn is an integral part of ALP, and its activity could serve as a marker of Zn nutritional status [39,40]. In the current study, the concentration of serum ALP increased with the two supplementary Zn sources at 14 days of age, which is in agreement with Ma et al. [41], who reported that supplementary Zn methionine and ZnO increased serum ALP and MT concentrations in calves. 

In the current study, the plasma Zn concentrations in ZnPro and ZnO calves were higher than in the CON calves, which indicates that the addition of Zn is beneficial for the absorption of Zn. It is known that plasma Zn concentration is closely related to dietary Zn intake and that serum zinc increased with Zn supplementation [42]. Wright and Spears [43] reported an increase in plasma Zn concentration in Holstein calves following dietary zinc administration, while Wang et al. [44] reported that ZnPro increased plasma Zn concentration in lactating cows. Similar results were observed in a previous study by Wo et al. [17], who found that supplementation with ZnPro increased the serum Zn concentration in dairy calves. However, Zn supplementation did not affect the plasma concentrations of other trace elements (Ca, Cu, Fe, Mg, and P).

## 5. Conclusions

Supplementation with ZnPro reduced the incidence of diarrhea and promoted the growth performance by increasing the ADG and decreasing the FGR in Holstein dairy calves during their first 28 days of life. ZnO supplementation showed similar effects to ZnPro, but it only exhibited a growth-promoting effect during days 1 to 14 and an anti-diarrheal effect during days 15 to 28 and 1 to 28. ZnPro improved serum IgG and IgM concentrations on d 14 and d 28, whereas ZnO improved serum IgG and IgM concentrations only at d 28. Furthermore, ZnO increased the serum concentration of IL-10 on d 14, but decreased that of IL-1β on d 28. The different Zn sources elevated the serum ALP and MT concentrations at 14 days of age and plasma Zn concentration during the whole experimental period. ZnPro improved the antioxidant capacity in the Holstein dairy calves to a greater extent than ZnO. The present study indicates that ZnPro has the potential to be an organic source of zinc and can be used as an anti-diarrheal and growth promoting agent for dairy calves during their early life.

## Figures and Tables

**Table 1:** Nutrient composition of the milk.

Items	Milk
Milk protein, %	3.77
Milk fat, %	4.80
Total solids, %	14.2
SNF, %	9.43
Lactose, %	4.97
Density, g/mL	1.04

SNF: solids-non-fat.

**Table 2:** Nutrient composition of the starter.

Items	Starter (DM Basis)
CP, %	25.0
Ether extract, %	3.41
Ash, %	6.23
NDF, %	25.7
ADF, %	17.0
DM, %	89.5
Zinc, mg/kg	83.9

CP: crude protein; NDF: neutral detergent fiber; ADF: acid detergent fiber; DM: dry matter.

**Table 3:** Effects of supplementary zinc proteinate (ZnPro) and zinc oxide (ZnO) on average daily gain and incidence of diarrhea in Holstein calves ^1^.

Items	CON	ZnPro	ZnO	SEM	*p*-Value
Initial Body Weight, kg	42.5	41.4	42.6	1.92	0.886
1–14 days of age					
ADG (g/d)	536 ^b^	633 ^a^	612 ^a^	22.6	0.017
Starter DMI (g/d)	41.2	47.5	46.0	9.06	0.877
Total DMI (g/d)	1151	1157	1155	9.06	0.877
FGR	2.18 ^a^	1.84 ^b^	1.89 ^b^	0.08	0.018
Incidence of diarrhea (%)	25.9 ^a^	10.7 ^b^	16.1 ^a, b^	-	0.012
15–28 days of age					
ADG (g/d)	723	862	796	43.3	0.097
Starter DMI (g/d)	73.8	78.8	89.4	14.0	0.728
Total DMI (g/d)	1406	1411	1421	14.0	0.728
FGR	1.88	1.65	1.82	0.08	0.136
Incidence of diarrhea (%)	24.1 ^a^	8.04 ^b^	11.6 ^b^	-	0.003
1–28 days of age					
ADG (g/d)	630 ^b^	747 ^a^	706 ^a, b^	26.3	0.017
Starter DMI (g/d)	57.5	63.2	67.7	9.97	0.772
Total DMI (g/d)	1278	1284	1289	9.97	0.772
FGR	2.02 ^a^	1.75 ^b^	1.85 ^a, b^	0.07	0.023
Incidence of diarrhea (%)	25.0 ^a^	9.38 ^b^	13.8 ^b^	-	<0.001

^a, b^ Means in the same row with different superscripts differ significantly from each other (*p* < 0.05). ^1^ Data are presented as means with SEM (n = 8). CON: control; ZnPro: 627 mg/d ZnPro (80 mg zinc/day); ZnO: 101 mg/d ZnO (80 mg zinc/d). ADG: average daily gain; DMI: dry matter intake; FGR: feed:gain ratio.

**Table 4:** Effects of supplementary zinc proteinate (ZnPro) and zinc oxide (ZnO) on serum immune function of Holstein newborn calves ^1^.

Items	CON	ZnPro	ZnO	SEM	*p*-Value
14 days of age					
IgM (mg/mL)	4.85 ^b^	5.41 ^a^	5.50 ^a^	0.10	<0.001
IgA (µg/mL)	30.4	30.2	30.5	0.54	0.898
IgG (mg/mL)	8.29 ^b^	9.05 ^a^	9.12 ^a^	0.19	0.014
IFN-γ (pg/mL)	117	123	116	2.98	0.201
IL-10 (pg/mL)	186 ^b^	200 ^a, b^	216 ^a^	7.44	0.034
IL-1β (pg/mL)	243	235	232	5.04	0.287
28 days of age					
IgM (mg/mL)	5.29 ^b^	5.76 ^a^	5.68 ^a, b^	0.12	0.021
IgA (µg/mL)	30.8	30.2	30.5	0.88	0.749
IgG (mg/mL)	8.85 ^b^	9.65 ^a^	9.58 ^a^	0.20	0.015
IFN-γ (pg/mL)	117	122	119	5.78	0.800
IL-10 (pg/mL)	173	182	177	8.09	0.744
IL-1β (pg/mL)	219 ^a^	201 ^a, b^	191 ^b^	5.78	0.007

^a, b^ Mean values in the same row with different superscripts are significantly different (*p* < 0.05). ^1^ Data are presented as means with SEM (n = 8). CON: control; ZnPro: 627 mg/d ZnPro (80 mg zinc/day); ZnO: 101 mg/d ZnO (80 mg zinc/d). IgM: immunoglobulin M; IgA: immunoglobulin A; IgG: immunoglobulin G; IFN-γ: interferon-γ; IL-10: interleukin-10; IL-1β: interleukin-1β.

**Table 5:** Effects of supplementary zinc proteinate (ZnPro) and zinc oxide (ZnO) on serum antioxidant concentrations in Holstein newborn calves ^1^.

Items	CON	ZnPro	ZnO	SEM	*p*-Value
14 days of age					
MDA (nmol/mL)	3.41 ^a^	3.05 ^b^	3.20 ^a, b^	0.09	0.037
SOD (U/mL)	50.4	59.0	58.4	0.33	0.124
T-AOC (mmol)	0.17 ^b^	0.20 ^a^	0.21 ^a^	0.01	0.002
28 days of age					
MDA (nmol/mL)	3.25 ^a^	2.81 ^b^	2.87 ^b^	0.12	0.027
SOD (U/mL)	53.7	55.8	60.3	0.38	0.471
T-AOC (mmol)	0.16	0.16	0.17	0.01	0.779

^a, b^ Means in the same row with different superscripts are significantly different from each other (*p* < 0.05). ^1^ Data are presented as means with SEM (n = 8). CON: control; ZnPro: 627 mg/d ZnPro (80 mg zinc/day); ZnO: 101 mg/d ZnO (80 mg zinc/day). SOD: superoxide dismutase; T-AOC: total antioxidant capacity; MDA: malondialdehyde.

**Table 6:** Effects of supplementary zinc proteinate (ZnPro) and zinc oxide (ZnO) on concentrations of serum zinc-dependent protein in Holstein newborn calves ^1^.

Items	CON	ZnPro	ZnO	SEM	*p*-Value
14 days of age					
ALP (pg/mL)	1852 ^b^	2041 ^a^	1994 ^a^	45.6	0.021
MT (pg/mL)	909 ^b^	994 ^a^	1003 ^a^	25.0	0.027
28 days of age					
ALP (pg/mL)	1811	1735	1817	48.7	0.428
MT (pg/mL)	856	921	881	31.9	0.362

^a, b^ Means in the same row with different superscripts are significantly different from each other (*p* < 0.05). ^1^ Data are presented with the means and SEM (n = 8). CON: control; ZnPro: 627 mg/d ZnPro (80 mg zinc/day); ZnO: 101 mg/d ZnO (80 mg zinc/d). ALP: alkaline phosphatase; MT: metallothionein.

**Table 7:** Effects of supplementary zinc proteinate (ZnPro) and zinc oxide (ZnO) on plasma trace element concentrations in Holstein calves ^1^.

Items	CON	ZnPro	ZnO	SEM	*p*-Value
14 days of age					
Zn (mg/L)	1.32 ^b^	2.08 ^a^	1.95 ^a^	0.19	0.023
Ca (mg/L)	92.5	101	95.9	4.35	0.399
Cu (mg/L)	0.76	0.77	0.76	0.04	0.983
Fe (mg/L)	1.89	1.81	2.17	0.49	0.865
Mg (mg/L)	17.0	17.6	16.5	0.65	0.559
P (mg/L)	126	120	121	5.03	0.637
28 days of age					
Zn (mg/L)	1.20 ^b^	1.96 ^a^	1.81 ^a^	0.16	0.011
Ca (mg/L)	91.4	95.1	92.6	1.46	0.200
Cu (mg/L)	0.75	0.75	0.77	0.05	0.954
Fe (mg/L)	1.69	1.63	2.05	0.45	0.783
Mg (mg/L)	15.1	15.3	16.1	0.68	0.529
P (mg/L)	119	125	121	6.60	0.768

^a, b^ Means in the same row with different superscripts are significantly different from each other (*p* < 0.05). ^1^ Data are presented as means with SEM (n = 8). CON: control; ZnPro: 627 mg/d ZnPro (80 mg zinc/day); ZnO: 101 mg/d ZnO (80 mg zinc/d).

## Data Availability

Data are contained within the article.
